# Unexpected peritonitis: Spontaneous gallbladder perforation without prior cholecystitis in an elderly patient - A case report

**DOI:** 10.1016/j.ijscr.2025.111691

**Published:** 2025-07-24

**Authors:** Bijay Raj Bhatta, Samrat Shrestha, Mecklina Shrestha, Sabin K. Ghimire, Rabin K. Ghimire

**Affiliations:** a National Academy of Medical Sciences, NAMS, Bir Hospital, Department of General Surgery, Kathmandu, Nepal; b College of Medical Sciences(CoMS), Department of Pathology, Bharatpur, Nepal; c National Academy of Medical Sciences, NAMS, Bir Hospital, Department of Radiodiagnosis, Kathmandu, Nepal

**Keywords:** Gallbladder, Perforation, Acute abdomen, Cholecystectomy, Niemeier classification, Cholecystitis, Case report

## Abstract

**Introduction and importance:**

Gallbladder perforation is a rare but serious complication, usually following acute cholecystitis. Spontaneous perforation without prior biliary symptoms is extremely uncommon and often presents as acute peritonitis, especially in the elderly and/or immunocompromised patients. Early diagnosis and prompt surgical intervention are essential to reduce morbidity and mortality.

**Case presentation:**

A 76-year-old male with no prior history of gallbladder disease presented with a 3-day history of right upper quadrant pain and signs of generalized peritonitis. Imaging revealed a perforation in the gallbladder fundus with pericholecystic fluid and intraluminal stones. Emergency laparotomy confirmed a 5 mm fundal perforation with 500 ml of bile-stained peritoneal fluid. Cholecystectomy and peritoneal lavage were performed. The patient recovered uneventfully.

**Clinical discussion:**

Spontaneous gallbladder perforation is rare, especially without prior cholecystitis. It most commonly occurs at the fundus due to its limited blood supply, making it susceptible to ischemia. While cholecystitis is more frequent in females, perforation is more common in elderly males. Clinical signs may be nonspecific, and perforation is often diagnosed only via imaging or intraoperatively. Contrast-enhanced computed tomography is the most sensitive imaging modality, identifying mural defects, pericholecystic fluid, and fat stranding. Laparoscopic or open cholecystectomy with peritoneal lavage remains the mainstay of treatment.

**Conclusion:**

Spontaneous gallbladder perforation should be considered in elderly patients with an acute abdomen, even without a history of gallbladder disease. Timely imaging and surgical management are vital for favorable outcomes. Early recognition, appropriate classification, and individualized treatment planning can significantly reduce complications and improve long-term prognosis.

## Highlights

• Spontaneous gallbladder perforation without prior cholecystitis is rare, symptoms are nonspecific, often in elderly males.
• Gallbladder fundus is the commonest site for perforation, leads to Type 1 (Niemeier) perforation with biliary peritonitis.
• Contrast-enhanced computed tomography has high accuracy than ultrasound in detecting mural defects and pericholecystic fluid.
• Emergency open cholecystectomy is preferred for Type 1 perforation, especially with bile peritonitis and sepsis.
• Laparoscopic or percutaneous drainage may be options in stable patients, but delayed cholecystectomy is often needed.

## 1 Introduction

Gallbladder (GB) perforation is a known but rare complication of acute cholecystitis, whether it is calculous or acalculous cholecystitis. The incidence of acute cholecystitis with gallbladder perforation has been reported in the range of 2 to 18 % [1]. There have also been a few cases of spontaneous GB perforation without prior history of cholecystitis [2,3]. Most of the time, adult to elderly age group patients present with right upper quadrant pain, nausea/vomiting, abdominal distention, and signs of peritonitis [4]. Clinical suspicion of GB perforation is often proved by the help of radiology, with contrast-enhanced computed tomography (CECT) scan being more accurate than ultrasonography of the abdomen in this matter [5]. Occasionally, diagnosis is made intraoperatively while performing laparoscopy or laparotomy for generalized peritonitis. We are reporting the case of an elderly male with spontaneous GB perforation with no prior history of cholecystitis and other comorbidities. This case has been reported in line with the revised SCARE guidelines, 2025 [6].

## 2 Case presentation

A 76-year-old male presented to the emergency department of our hospital with complaints of progressively worsening abdominal pain for the past 3 days. The pain was acute in onset, localized to the right upper quadrant, and was described as constant, dull aching, and non-radiating. It was associated with mild nausea but no episodes of vomiting, jaundice, or fever. He had no recent history of abdominal trauma. The patient was a chronic smoker. Bowel and bladder habits were normal. The patient had no significant past medical history, including no known comorbidities such as diabetes, hypertension, or liver disease. He had no prior history of similar abdominal pain, gallstones, cholecystitis, or any abdominal surgeries.

On examination, the patient was afebrile, pulse rate was 105 beats/min, blood pressure was 110/70 mmHg, but appeared mildly dehydrated and icteric. Abdominal examination revealed generalized tenderness and guarding, more to the right upper quadrant, with signs of peritonitis. There was no palpable mass, organomegaly, or visible distension. Laboratory investigations showed a mildly elevated white blood cell count (13,200/μl) with neutrophilic predominance (Neutrophil-90 %). Liver function tests showed total bilirubin: 3.1 mg/dl and direct bilirubin: 0.8 mg/dl, alanine aminotransferase (ALT): 22 IU/L; aspartate aminotransferase (AST): 41 IU/L; alkaline phosphatase: 122 IU/L; total protein: 6.1 g/dl and albumin: 3 g/dl. The rest of the parameters were within normal limits.

**Fig. 1:**
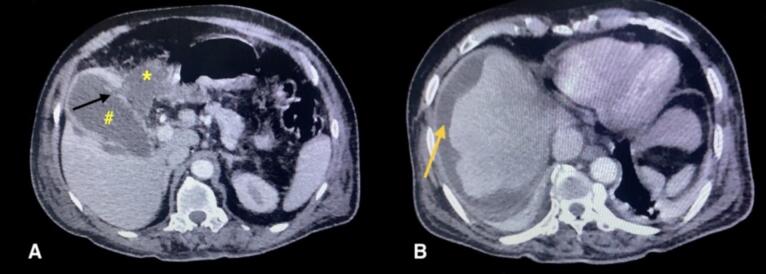
CECT abdomen axial section demonstrating **Panel A:** Gallbladder perforation at the fundus, indicated by a black arrow. A pericholecystic fluid collection is noted and marked with an asterisk (*). The gallbladder is marked with a hash symbol (#). **Panel B:** Perihepatic fluid collection is visualized and marked by a yellow arrow. CECT: Contrast-enhanced computed tomography. (For interpretation of the references to colour in this figure legend, the reader is referred to the web version of this article.)

Initial abdominal ultrasonography revealed a mildly distended gallbladder with pericholecystic fluid with multiple calculi in the gallbladder lumen. However, there was no biliary duct dilation. A subsequent CECT scan of the abdomen revealed a focal defect of approximately 4 mm in the gallbladder fundus with a pericholecystic fluid collection (approximately 80 ml), suggestive of gallbladder perforation (Fig. 1). Few calculi were noted within the gallbladder lumen, the largest being 7 × 6 mm. Based on imaging findings and clinical presentation, a diagnosis of spontaneous gallbladder perforation (Niemeier type 1) was made.

**Fig. 2:**
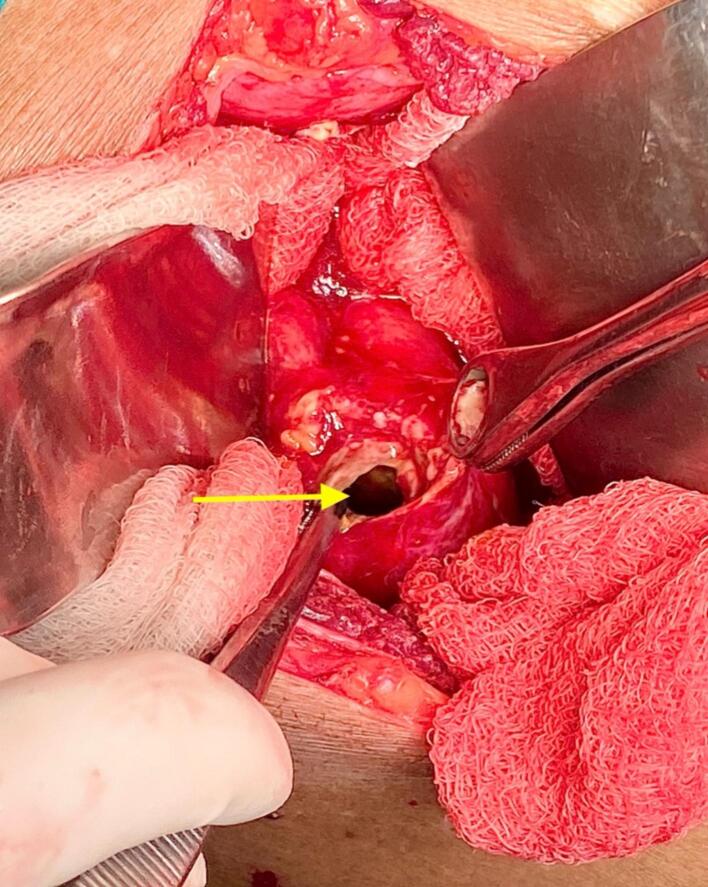
Intraoperative image demonstrating perforation at the fundus of the gallbladder (marked by yellow arrow). The perforation site appears as a well-defined defect in the gallbladder wall, with surrounding inflamed and edematous tissue. (For interpretation of the references to colour in this figure legend, the reader is referred to the web version of this article.)

**Fig. 3:**
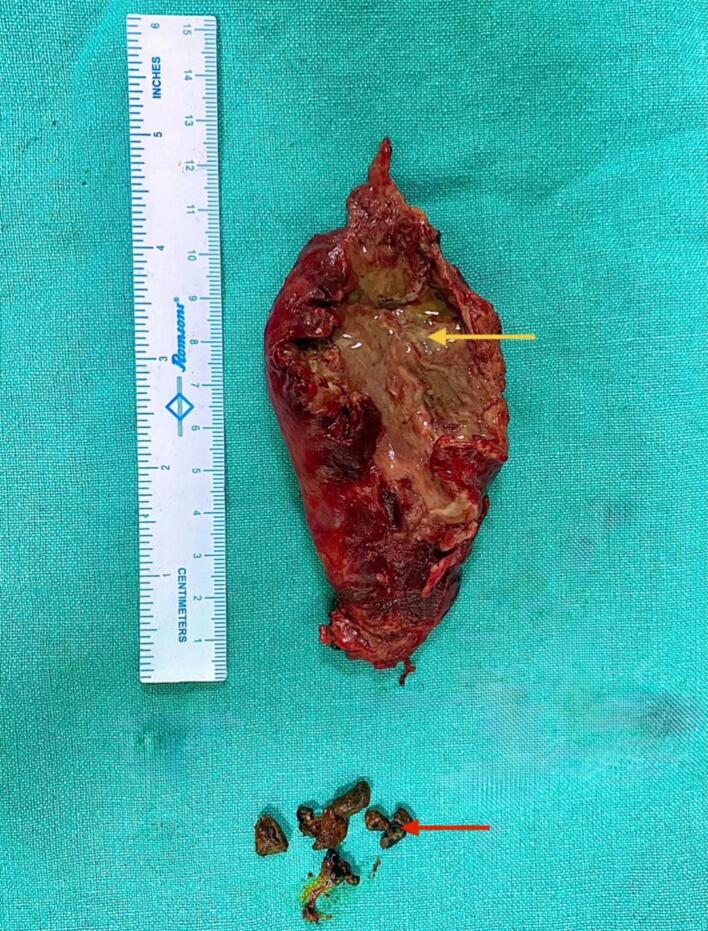
Gross specimen of the excised gallbladder following cholecystectomy. The gallbladder has been cut open to reveal the internal pathology. The yellow arrow indicates the area of fundal perforation with necrotic and gangrenous mucosal lining. The red arrow highlights a gallstone extracted from the gallbladder lumen. (For interpretation of the references to colour in this figure legend, the reader is referred to the web version of this article.)

The patient underwent an emergency exploratory laparotomy, which revealed approximately 500 ml of bile-stained peritoneal fluid. A 5 mm perforation was identified at the fundus of the gallbladder (Fig. 2), with dense adhesions between the gallbladder, liver, and omentum at the site of perforation. Surrounding structures, including the duodenum, were carefully inspected and found to be normal. Cholecystectomy with thorough peritoneal lavage and drain placement was performed. The gallbladder was cut open to reveal the internal pathology. Fundal perforation with necrotic and gangrenous mucosal lining was noted. Gallstone was extracted from the gallbladder lumen (Fig. 3). In the postoperative period, he received third-generation cephalosporin and metronidazole via the intravenous route for 5 days, which were also supported by culture and sensitivity report (Bile culture revealed growth of *E. coli*, sensitive to third-generation cephalosporin in combination with metronidazole to provide additional anaerobic coverage). The postoperative course was uneventful, and the patient was discharged on postoperative day 5 in a stable condition. Histopathological examination of the gallbladder revealed chronic inflammation and an area of acute inflammation with focal necrosis and no evidence of malignancy. The patient expressed surprise at the severity of his condition, having never experienced prior gallbladder issues. He was grateful for the timely diagnosis and surgical care, and reported feeling significantly better within days of the operation.

The patient was regularly followed up in the outpatient department after discharge. At 2-week and 1-month follow-up visits, he remained asymptomatic with no signs of wound infection, jaundice, or abdominal complaints. Laboratory investigations, including liver function tests and complete blood counts, were within normal limits. On subsequent follow-up at 3 months and 6 months, the patient remained asymptomatic.

## 3 Discussion

Gallbladder perforation is a rare phenomenon associated with high morbidity and mortality and clinicians must remain alert to GB perforation in elderly patients, even in the absence of fever or classic signs of peritonitis. Most of the time, it follows the episode of calculous or acalculous cholecystitis. Acute cholecystitis resulting in GB perforation has been reported in various articles, with incidence ranging from 2 % - 18 % [1]. GB perforation without prior history of cholecystitis has been termed as spontaneous GB perforation. It is an even more rare condition [2,3]. Our patient presented with right upper quadrant abdominal pain without systemic signs of infection or a prior history of gallbladder disease, which made initial clinical suspicion of GB perforation less likely**.** Common etiologies behind the spontaneous GB perforation are ischemia, infection, and cholelithiasis. The fundus of the GB is the most common site of perforation (60 % of the cases) because of poor blood flow, as the cystic artery being the end artery. Although cholecystitis is more common in females, the rate of GB perforation is higher in males, with the ratio of 3:2 [4]. This aligns with our case of a 76-year-old male, who had no known comorbidities but still developed this severe complication, indicating that age alone can be a predisposing factor. Elderly males with comorbidities and immunocompromised status are the usual victims of GB perforation, but there have been few reported cases where adolescents and even neonates have suffered from this condition. [7,8]

In 1934, Niemeier classified GB perforation into 3 types [9]. Type 1 is an acute state, where there is free perforation and biliary peritonitis. Type 2 is characterized by a subacute presentation of local peritonitis and abscess formation as fluid is localized at the site of perforation. There is also a chance of fistula formation with the adjacent organ in the long run (cholecystoenteric fistula), which is classified as type 3. Type 2 is more common than the others, while type 1 is more lethal [10]. The presence of 500 ml of bile-stained fluid and a 5 mm defect in the gallbladder fundus during surgery confirmed a free perforation with generalized peritonitis, fitting Type I criteria. Another type, type 4, was introduced by Anderson et al. in 1984, which is characterized by the formation of cholecystobiliary fistula [11]. Another way to classify GB perforation is according to etiology: iatrogenic, traumatic, and spontaneous types as proposed by Estevao-Costa et al. [12] Common ways of presentation are either acute or subacute, with the features of generalized or localized peritonitis, respectively. Other rare clinical features are symptoms of acute gastroenteritis or those of cholecystoenteric fistula [1,13]. Although high-grade fever and leukocytosis are present in most cases of GB perforation, it is not diagnostic, as many cases of acute cholecystitis also have similar features. In our patient, early symptoms were nonspecific, with only mild nausea and no fever, which initially obscured the diagnosis.

The first radiological test in any gallbladder-related pathology would be an ultrasound of the abdomen, as in this case. The key features of GB perforation in the ultrasound are a defect in the GB wall/ sonographic hole sign, pericholecystic fluid collection, thickened or striated GB wall, and sometimes stones outside the GB [5]. CECT of abdomen has more sensitivity and diagnostic accuracy than USG of the abdomen to diagnose the case of GB perforation. The most specific and diagnostic finding in a CT scan is a mural defect in the GB wall; other features include GB wall thickening and enhancement, intramural abscess or gas, pericholecystic fluid collection, and fat strandings [14]. Other less commonly preferred second-line imaging techniques include Doppler ultrasound, magnetic resonance imaging, and radionuclide scans. Few cases are diagnosed intraoperatively after initial negative imaging tests in patients with localized or generalized peritonitis [15].

Treatment of the GB perforation depends on the type, timing of presentation, and severity of the patient at presentation. Most of the Niemeier type 1 and type 2 cases can be managed with emergency exploratory laparotomy with cholecystectomy, peritoneal lavage, and drain placement [16]. Given the volume of bile-stained fluid in the peritoneum and the clear perforation identified intraoperatively, open cholecystectomy was deemed the most appropriate surgical approach in our case. Laparoscopic technique is advisable in the presence of expertise, with many systematic reviews showing the advantage of laparoscopic surgery in terms of more successful drainage, sepsis control, and shorter hospital stay. In recent years, with increasing number of laparoscopic approach, the rate of need for another intervention is in decreasing trend, which favors laparoscopic technique over open surgical approach [17]. USG or CT-guided percutaneous drainage or cholecystostomy is a feasible option in patients with severe sepsis or multiple comorbidities with poor performance status, but this should be followed by cholecystectomy once the patient is stabilised [16]. Type 3 and 4 patients are difficult to manage because of the patient's condition and the complexity of the disease. These types often require reconstructive procedures along with repair of the fistula [18].

## 4 Conclusion

This case emphasizes that gallbladder perforation, although a rare complication of acute cholecystitis, warrants prompt recognition and intervention. Even in the absence of prior symptomatic cholecystitis, spontaneous perforation can occur, often precipitated by gallstones causing focal ischemia, underscoring the need to consider this diagnosis in any patient presenting with acute right upper quadrant pain and signs of peritonitis. CECT remains the most sensitive modality, facilitating preoperative planning. Employing Niemeier's classification helps guide surgical strategy: Type I lesions, characterized by free perforation and generalized biliary peritonitis, mandate immediate surgery, open or laparoscopic, whereas selected Type II and III cases may be amenable to more conservative or delayed approaches in experienced hands. Given the significant risk associated with delayed management, clinicians should maintain a low threshold for advanced imaging and surgical consultation in elderly or high-risk patients, even in the absence of classic cholecystitis histories.

## Consent

Written informed consent was obtained from the patient for publication of this case report and accompanying images. A copy of the written consent is available for review by the Editor-in-Chief of this journal on request.

## Ethical approval

Ethical approval is waived at our institution and this study was exempt from ethical approval at our institution, as this paper reports a single case that emerged during a normal surgical case report.

## Funding

This research did not receive any specific grant from funding agencies in the public, commercial, or not-for-profit sectors.

## Author contribution

1. Constructing hypothesis for the manuscript- Samrat Shrestha, Bijay Raj Bhatta.
2. Planning methodology to reach the conclusion: Samrat Shrestha, Bijay Raj Bhatta, Mecklina Shrestha.
3. Organizing and supervising the course of the article and taking responsibility: Samrat Shrestha.
4. Patient follow-up and reporting – Mecklina Shrestha, Bijay Raj Bhatta, Sabin K Ghimire, Rabin K. Ghimire
5. Logical interpretation and presentation of the results- Bijay Raj Bhatta, Samrat Shrestha, Mecklina Shrestha, Sabin K Ghimire, Rabin K. Ghimire.
6. Construction of the whole or body of the manuscript- Bijay Raj Bhatta, Samrat Shrestha, Mecklina Shrestha, Rabin K. Ghimire and Sabin K Ghimire.
7. Reviewing the article before submission not only for spelling and grammar but also for its intellectual content- Samrat Shrestha, Bijay Raj Bhatta, Mecklina Shrestha, Rabin K. Ghimire and Sabin K Ghimire.

## Guarantor

Samrat Shrestha accepts full responsibility for the work and/or the conduct of the study, has access to the data, and controls the decision to publish.

## Research registration number

1. Name of the registry: Zenodo

2. Unique identifying number or registration ID: 10.5281/zenodo.16226251

3. Hyperlink to your specific registration (must be publicly accessible and will be checked): https://doi.org/10.5281/zenodo.16226251

## Conflict of interest statement

No conflict of interest.
